# Eosinophilic pulmonary disease with ulcerative colitis: A case report

**DOI:** 10.1097/MD.0000000000037851

**Published:** 2024-04-26

**Authors:** Songtao Li, Mingliang Fang, Hua Guo, Tian Yu, Jun Wang, Weiming Li, Yunong Huang, Xichuan Wang, Rongyi Wu

**Affiliations:** a Department of Respiratory and Critical Care Medicine, The 6th People’s Hospital of Chengdu, Chengdu 610051, China; b Department of Cardiovascular Medicine, The second People’s Hospital of Chengdu, Chengdu 610017, China; c Department of Radiology, The 6th People’s Hospital of Chengdu, Chengdu 610051, China; d Department of Pathology, The 6th People’s Hospital of Chengdu, Chengdu 610051, China.

**Keywords:** case report, eosinophilic lung disease, ulcerative colitis

## Abstract

**Rationale:**

Eosinophilic pulmonary disease (EPD) is a general term for a large group of diseases with complex etiology. Ulcerative colitis is an inflammatory bowel disease (IBD). Patients with IBD may have pulmonary involvement. We herein present a case of ulcerative colitis complicated with EPD.

**Patient concerns:**

A 34-year-old woman with ulcerative colitis presented with dry cough. She had peripheral eosinophilia and apical ground glass opacities on CT (computed tomography) of her chest. Antibiotic treatment was ineffective.

**Diagnoses:**

Lung biopsy revealed eosinophil infiltration in the alveolar space and interstitial space, so EPD was considered.

**Interventions:**

After oral administration of prednisone, the lung shadow on CT disappeared when the cough symptoms resolved. However, the symptoms recurred after drug withdrawal, and the lung shadow reappeared on imaging. The cough symptoms and lung shadow disappeared after oral prednisone was given again. Prednisone was slowly discontinued after 6 months of treatment.

**Outcomes:**

The patient stopped prednisone for half a year. No recurrence or abnormal CT findings were detected during the half-year follow-up.

**Lessons:**

The clinical manifestations of EPD are atypical, laboratory and imaging findings are not specific, and it is difficult to make a definite diagnosis before lung biopsy. The diagnosis depends on pathological examination. Glucocorticoid treatment is effective, but some patients may relapse after drug withdrawal. Active follow-up after glucocorticoid treatment is very important for identifying disease recurrence. Patients with IBD are relatively prone to developing EPD. The etiology of EPD is complex. In clinical practice, we need to make a diagnosis and differential diagnosis to clarify its etiology.

## 1. Introduction

Eosinophilic pulmonary disease (EPD) refers to airway and/or lung pleura cavity acidophilic granulocytes that increase the heterogeneity of a set of clinical diseases characterized by or without increased peripheral blood eosinophilia.^[1]^ EPD, which can be an isolated disease in the lung or a pulmonary manifestation of systemic disease, is a difficult problem in the diagnosis and treatment of respiratory diseases. The etiology of EPD is complex. The treatment of EPD may differ for different causes, and we need to carry out comprehensive and detailed etiological screening in clinical practice. A case of eosinophilic lung disease with ulcerative colitis is reported below.

## 2. Case presentation

**Figure 1.**
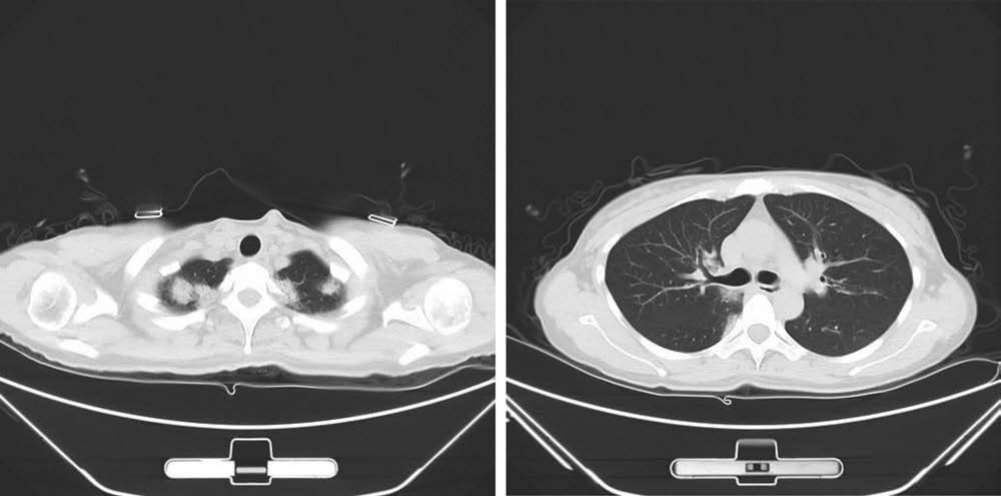
CT of the chest revealed multiple patchy opacities in both lungs.

**Figure 2.**
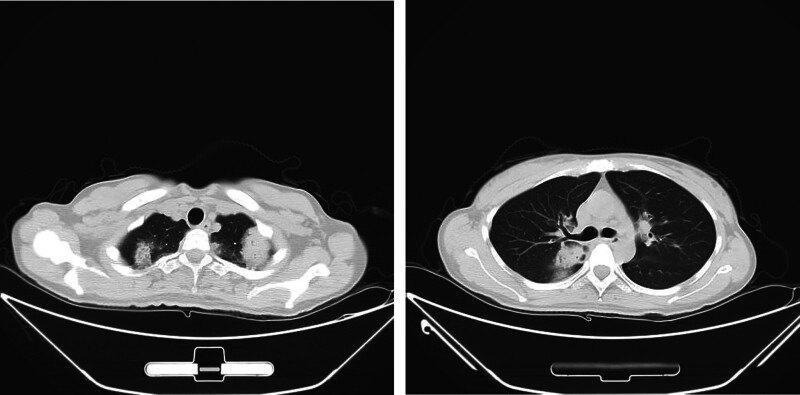
A repeat CT scan showed further expansion of the patchy lung shadow.

**Figure 3.**
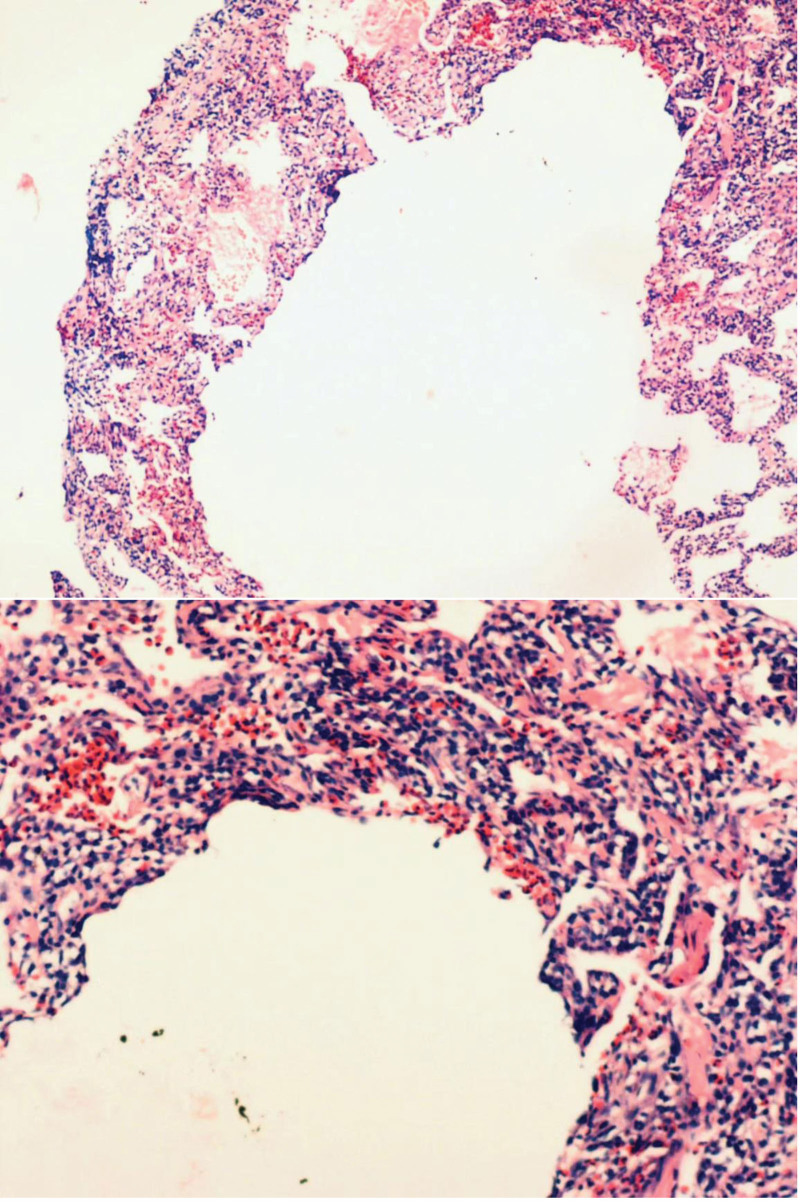
Pathological examination revealed extensive eosinophil infiltration in the alveolar space and alveolar interstitium, as determined by hematoxylin–eosin staining. (A) Original magnification ×20. (B) Original magnification ×40.

**Figure 4.**
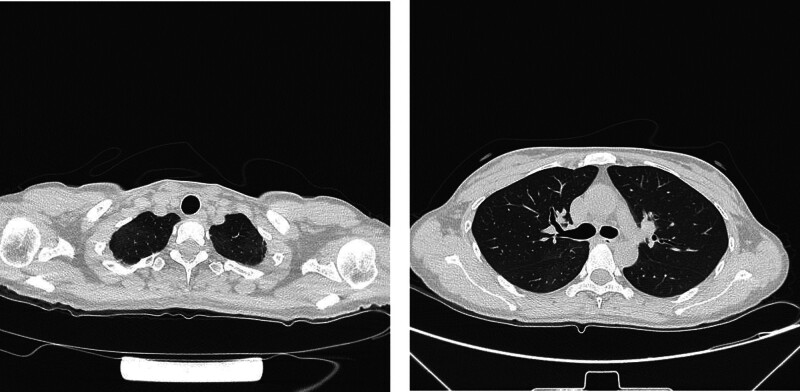
CT scan showing that the lung lesions were completely absorbed after 10 d of glucocorticoid treatment.

**Figure 5.**
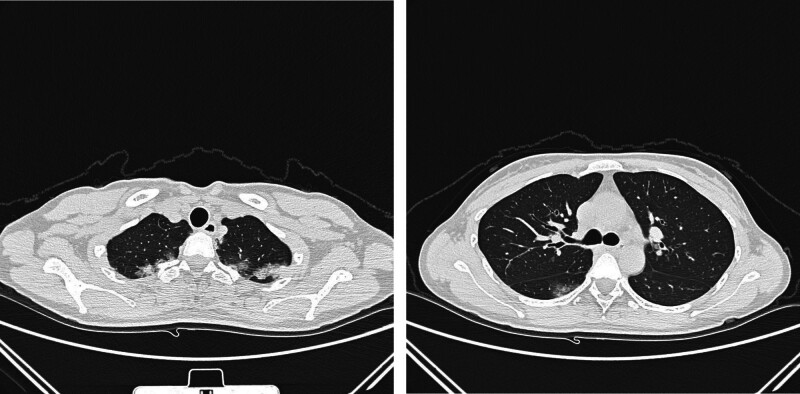
Chest CT showed bilateral patchy shadows in the lung, and the lesions were unchanged as compared to 6 mo prior.

**Figure 6.**
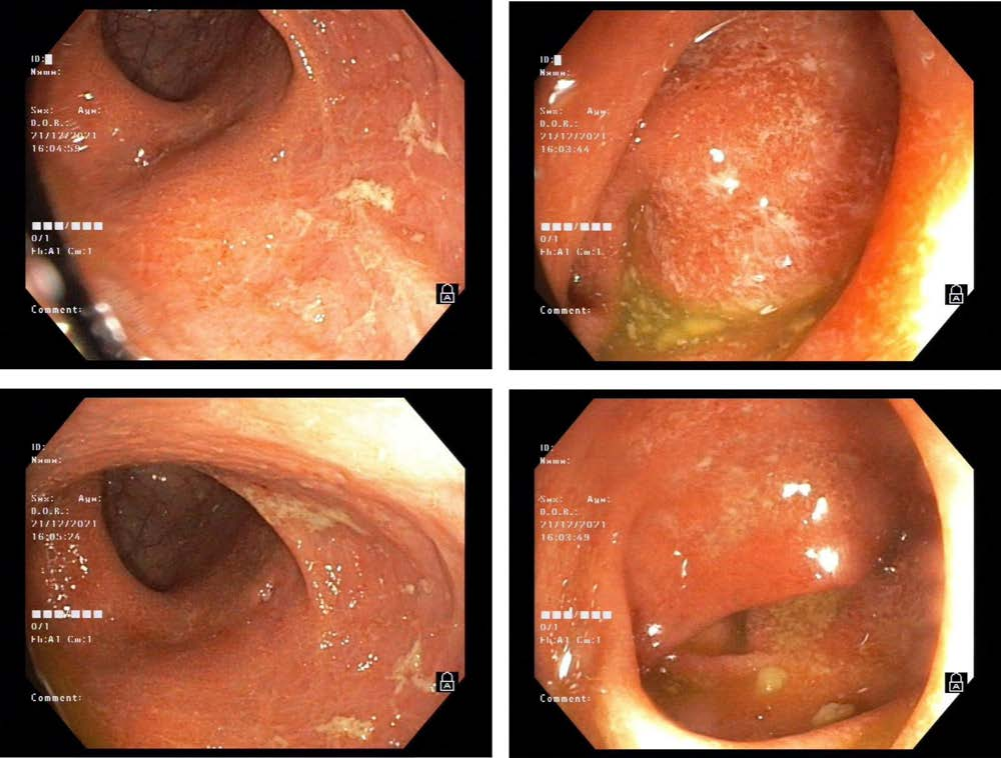
Colonoscopy revealed a small superficial ulcer.

**Figure 7.**
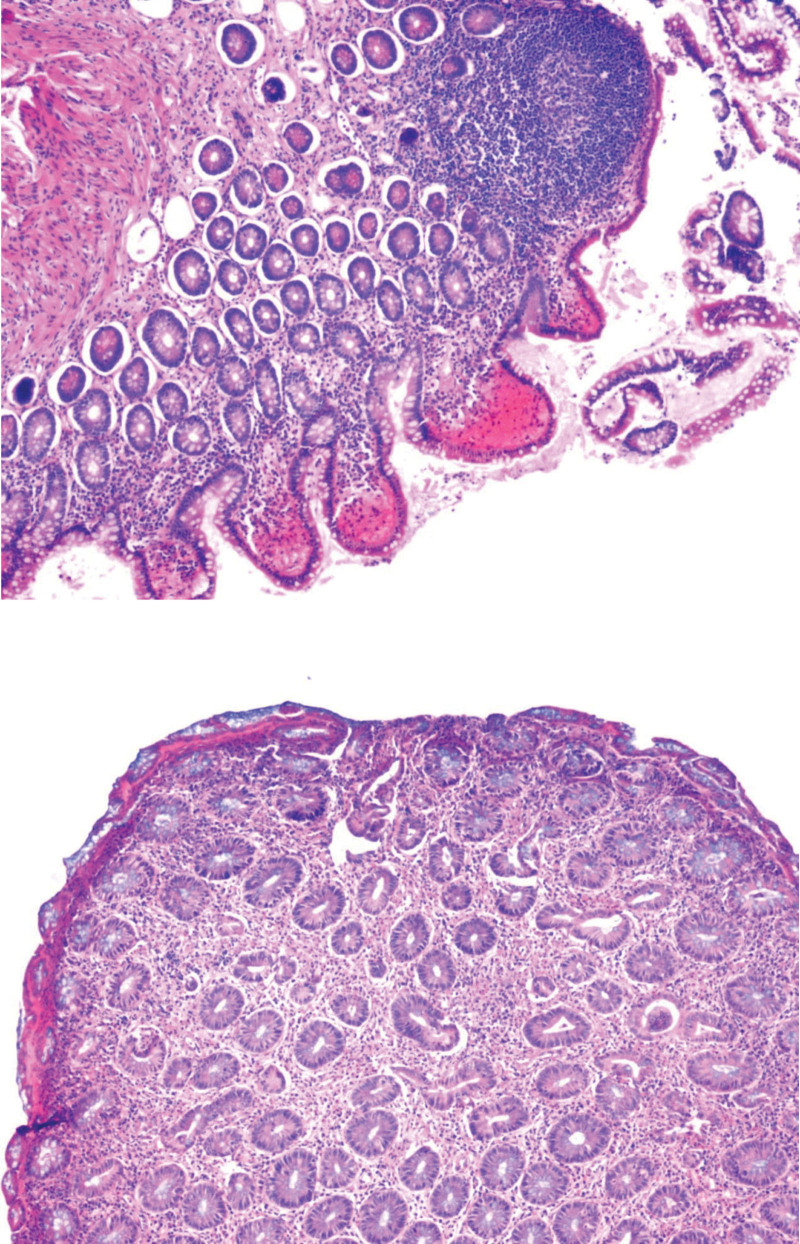
Colonoscopy biopsy revealed chronic active inflammation of the sigmoid colon mucosa with adenomatous hyperplasia in some glands; there was chronic active inflammation of the rectal mucosa with lymphoid follicle formation, as shown by hematoxylin–eosin staining. (A) Original magnification ×20. (B) Original magnification ×40.

A 34-year-old woman was admitted to the hospital on April 29, 2021, due to cough for 20 days. Twenty days before admission, the patient developed a cough, which was a dry and severe cough, but the patient had no fever, dyspnea, or hemoptysis. The patient was diagnosed with “ulcerative colitis” 5 years prior and was intermittently treated with “mesalazine,” which had a good effect. The patient complained of spontaneous relief of loose stools after pregnancy 2 years prior, and mesalazine was discontinued. 1 month before admission, her symptoms recurred, and “mesalazine” was used again to resolve the diarrhea. The patient was born and living in Sichuan, China, and denied a history of eating raw shrimp and crabs. The physical examination at admission revealed the following: T, 36.7°C; P, 78 bpm; R20, bpm; BP, 101/60 mm Hg; and SpO_2_, 97% (without oxygen inhalation). Her breathing was still steady, and her answers were pertinent. There was no ecchymosis or petechiae on the skin, no palpable enlarged lymph nodes, and no dry–wet rales in either lung. Her heart rhythm was normal, there was no murmur in the valve areas, and her abdomen was soft with no tenderness, rebound pain, or muscle tension. There was no pain in the liver or kidney, the muscle strength or muscle tone of the extremities was normal, and there was no edema in the lower limbs. Routine blood tests at admission revealed a white blood cell count of 7.68 × 10^9^/L, an eosinophil count of 0.87 × 10^9^/L↑, an eosinophil percentage of 11.3%↑, and a CRP level of 35.62 mg/L↑. The erythrocyte sedimentation rate was 71 mm/h↑, the results of routine urine and stool tests were normal, the results of urine and stool occult blood tests were negative, the PCT results were normal, and liver and kidney function, electrolytes, blood lipids, uric acid, and coagulation function were normal. Serum G and GM antigen tests, serum cryptococcal capsular antigen, tuberculosis antibody, PPD test (−), *Mycoplasma pneumoniae*, chlamydia antibody, and serum tumor markers were negative. CT of the chest revealed multiple patchy opacities in both lungs (Fig. 1). Empirical anti-infective treatment with cefotaxime was given. After treatment, the patient’s cough symptoms were not relieved, and routine blood reexamination on August 8, 2021, revealed a white blood cell count of 6.22 × 10^9^/L, an eosinophil count of 1.04 × 10^9^/L↑, an eosinophil percentage of 16.7%↑, and a CRP level of 13.64 mg/L↑. Her eosinophil count was greater than that at admission, and CT reexamination revealed that the scope of the patchy shadow in the lung was enlarged. Serum antinuclear antibody spectrum, ANCA and IgG4 were negative, and sinus CT showed no obvious abnormalities. There was no obvious abnormality on bronchoscopy, and the bacteria and fungi smear and culture of the lavage fluid were negative. The TB-DNA and GM tests of the lavage fluid were negative, and the exfoliative cytology of the lavage fluid was normal. mNGS of the lavage fluid was negative. On May 13, 2021, routine blood tests revealed white blood cells of 8.16 × 10^9^/L, eosinophils of 1.62 × 10^9^/L↑, eosinophils of 19.8%↑, and CRP of 32.35 mg/L↑. Her eosinophil count was greater than that at admission, and a repeat CT scan revealed further expansion of the patchy lung shadow (Fig. 2). No enlarged lymph nodes were found by color Doppler ultrasound of superficial lymph nodes in the whole body, and no definite masses were found by head, chest or abdomen enhanced CT. Gastroscopy revealed no definite abnormalities. Pulmonary function tests revealed an FVC of 2.55 L, an FVC of 76.9%, an FEV1/FVC of 94.29%, a DLCO of 101.9%, and increased airway resistance. CT-guided percutaneous lung biopsy was performed, and pathological examination revealed extensive eosinophil infiltration in the alveolar space and alveolar interstitium (Fig. 3), and acid-quickly, hexamine silver, and PAS staining was negative. Immunohistochemical analysis revealed CD20(+)p, CD3(+)p, Mum1(+), CD138(+), IgG4(less+, 10/HPF), S-100(−), CD1a(−), and Langerin(−) in plasma cells. Lymphocytes were negative for EBER1/2-ISH. EPD was considered. Prednisone 40 mg/d (0.75 mg/kg/d) was administered, the lung lesions were completely absorbed after 10 days of glucocorticoid treatment (Fig. 4), and the peripheral blood eosinophil counts returned to normal. The patient took oral glucocorticoids, and the dose was slowly reduced. During treatment with oral glucocorticoids, the patient’s ulcerative colitis did not flare, and mesalazine was not used. After 4 months of oral corticosteroid treatment, chest CT, routine blood tests and erythrocyte sedimentation rates were normal, and prednisone was discontinued. When the patient discontinued corticosteroids, her symptoms of mucopurulent and bloody stool recurred. Mesalazine was used again, and the symptoms were relieved. 3 months after drug withdrawal, the patient developed a cough again, a dry cough without sputum, accompanied by stool with no mucus, no chills, no dyspnea, and no hemoptysis. Routine blood examination showed that her eosinophil levels had increased again, chest CT showed bilateral patchy shadows in the lung, and the lesions were basically the same as those 6 months prior (Fig. 5). There were no abnormal findings on breast ultrasound or superficial lymph node ultrasound. A colonoscopy revealed a small superficial ulcer (Fig. 6), and a colonoscopy biopsy revealed chronic active inflammation of the ileocecal mucosa with focal hemorrhage in the superficial stroma. There was chronic active inflammation of the sigmoid colon mucosa with adenomatous hyperplasia in some glands; there was chronic active inflammation of the rectal mucosa with lymphoid follicle formation (Fig. 7). The possibility of recurrence of chronic eosinophilic pneumonia was considered. Oral glucocorticoids were given again, starting with 40 mg/d prednisone. After 2 weeks of treatment, chest CT reexamination revealed that the lesions were completely absorbed and resolved. The dose of prednisone was gradually reduced over a total of 6 months. In December 2022, she was followed up, and she did not have any coughing and was intermittently treated with mesalazine for ulcerative colitis.

## 3. Discussion

EPD is a general term for a group of diseases that can be diagnosed if any one of the following 4 criteria is met: pulmonary infiltration with increased peripheral blood EOS (>0.5 × 10^9^/L); increased EOS in bronchoalveolar lavage fluid (BALF) (>10%); EOS infiltration confirmed by surgical biopsy or transbronchial biopsy; or increased EOS in pleural effusion (≥10%).^[2–4]^ This patient had lung infiltration and increased peripheral blood eosinophils, so the diagnosis of EPD was confirmed. The etiology of EPD is complex and can be divided into secondary EPD with a definite etiology and idiopathic EPD with an unknown etiology. The etiology of secondary EPD includes the following: secondary infection, including parasites, viruses, bacteria, tuberculosis, fungi, etc.; allergic diseases, such as allergic bronchopulmonary aspergillosis and eosinophilic asthma; rheumatic diseases, such as eosinophilic granulomatosis with polyangiitis, IgG4-related disease, and Kimura disease; neoplastic diseases, such as hematological tumors and solid tumors; and other drugs, radiotherapy, transplantation, etc.^[1]^ Idiopathic EPD includes idiopathic acute eosinophilic pneumonia, idiopathic chronic eosinophilic pneumonia, primary simple pulmonary eosinophilia, idiopathic eosinophilic pleural effusion, and idiopathic hypereosinophilic syndrome.^[1]^ In terms of the etiology of this patient, infectious diseases, allergic diseases, and rheumatic immune diseases of secondary causes were not supported due to the combination of the patient’s symptoms and signs and the relevant laboratory, imaging, and pathological examinations. In neoplastic diseases and solid tumors, imaging examination revealed no definite space-occupying lesions, which did not support the possibility of solid tumors. However, persistent elevation of blood eosinophils has been reported in China during the treatment of polymyositis patients, and no cause was found after a detailed investigation. Approximately 3 years after the course of the disease, whole-body positron emission computed tomography was performed again, and metastatic lesions of the left axillary breast cancer were found.^[5]^ The eosinophil count of this patient returned to normal after steroid treatment, but the possibility of a tumor should be considered during long-term follow-up. In terms of other secondary causes, the patient had a history of ulcerative colitis and a personal history of mesalazine use. Cases of mesalazine-induced eosinophilic pneumonia have been reported.^[6–9]^ Lung injury caused by such drugs includes eosinophilic pneumonia, interstitial pneumonia, and fibrosing alveolitis,^[10]^ and the common clinical symptoms are dyspnea (76%), fever (68%), chest pain (65%), and cough (22%).^[11]^ The mechanism of mesalazine-induced lung injury is still unclear and may be related to the following factors: oxidative damage caused by the drug, direct cytotoxicity to alveolar capillary endothelial cells, and damage mediated by intracellular phospholipid deposition.^[12]^ However, in most cases, symptoms appear after 2 to 6 months of treatment. In this patient, mesalazine had been used for nearly 5 years, and when the EPD resolved, this patient was treated with mesalazine again for half a year, and there was no recurrence of EPD during follow-up. Therefore, mesalazine may not cause pulmonary involvement. The possibility of idiopathic EPD was considered. Considering the patient’s history, symptoms, and related laboratory and imaging findings, chronic eosinophilic pneumonia was considered a possibility. The main symptoms of chronic eosinophilic pneumonia are cough, dyspnea, fever and wheezing. There was an increase in eosinophils in the blood and BALF. The imaging manifestations are diffuse alveolar consolidation with a bronchial gas phase and/or ground glass shadow, especially peripheral distribution, indicating “reverse pulmonary edema,” and recurrence often occurs at the original site.^[13,14]^ The diagnostic criteria for chronic eosinophilic pneumonia were as follows: respiratory symptoms > 2 weeks; more EOS (>25%), eosinophilia in the blood and/or eosinophil infiltration in the lung; typical chest imaging findings; exclusion of other known causes of EPD (such as drugs and infection).^[13,14]^ The clinical features of this patient meet the above diagnostic criteria. Glucocorticoids are the main treatment drugs, and the usual initial medication and dose is prednisone 40 mg/d. After the symptoms improved and the lung lesions were absorbed, the dose was gradually reduced, and the course of treatment was 4 to 6 months. Patients are prone to relapse during the process of steroid reduction or withdrawal, but most respond well to steroid treatment again. The patient relapsed after drug withdrawal, and the recurrence site was basically the same as that at the first time. The effect of the second steroid treatment was good, which was consistent with previous reports. In summary, a diagnosis of chronic eosinophilic pneumonia was considered for this patient.

On the other hand, this patient had a clear history of inflammatory bowel disease (IBD). At present, cases of respiratory diseases related to IBD, which can involve the airways, pulmonary interstitials, and pulmonary vessels, have been reported at home and abroad. The pulmonary interstitials can manifest as eosinophilic pneumonia, which is sensitive to steroid treatment.^[15,16]^ The pathogenesis of pulmonary abnormalities caused by IBD is still unclear. At present, both the gastrointestinal tract and respiratory tract epithelium are believed to be derived from the primitive foregut, with columnar epithelium, goblet cells, submucosal mucous glands, and submucosal lymphoid tissue^[17]^; therefore, the lung damage caused by IBD is the inflammation of 2 different organs of the same embryonic origin. This high similarity triggers similar pathological changes. The underlying mechanism needs to be further studied. Further long-term follow-up is needed for the clinical treatment of this patient.

## 4. Conclusion

In summary, EPD is a rare disease with nonspecific clinical symptoms and a complex etiology, so the rates of misdiagnosis and missed diagnosis are high. In addition to infectious diseases, EPD should be considered in patients with underlying IBD if they have symptoms such as cough and dyspnea. IBD itself and certain drugs used to treat IBD can cause lung involvement. It is necessary for clinicians to actively evaluate imaging or even bronchoscopic BALF examination or lung biopsy for confirmation. After the diagnosis of EPD, it is necessary to further determine the cause and treat it. EPD may recur, and more than half a year of active follow-up is needed. A follow-up review is very important for identifying the recurrence of this disease. Of course, more comprehensive studies are needed on the etiology and pathogenesis of EPD to propose a standardized consensus on its diagnosis and treatment.

## Author contributions

**Conceptualization:** Songtao Li.

**Data curation:** Songtao Li.

**Project administration:** Songtao Li, Weiming Li.

**Writing—original draft:** Songtao Li.

**Writing—review & editing:** Songtao Li.

**Formal analysis:** Mingliang Fang.

**Methodology:** Mingliang Fang, Tian Yu, and Xichuan Wang.

**Software:** Mingliang Fang.

**Investigation:** Hua Guo, Yunong Huang.

**Resources:** Jun Wang, Xichuan Wang, and Rongyi Wu.

**Supervision:** Weiming Li.
